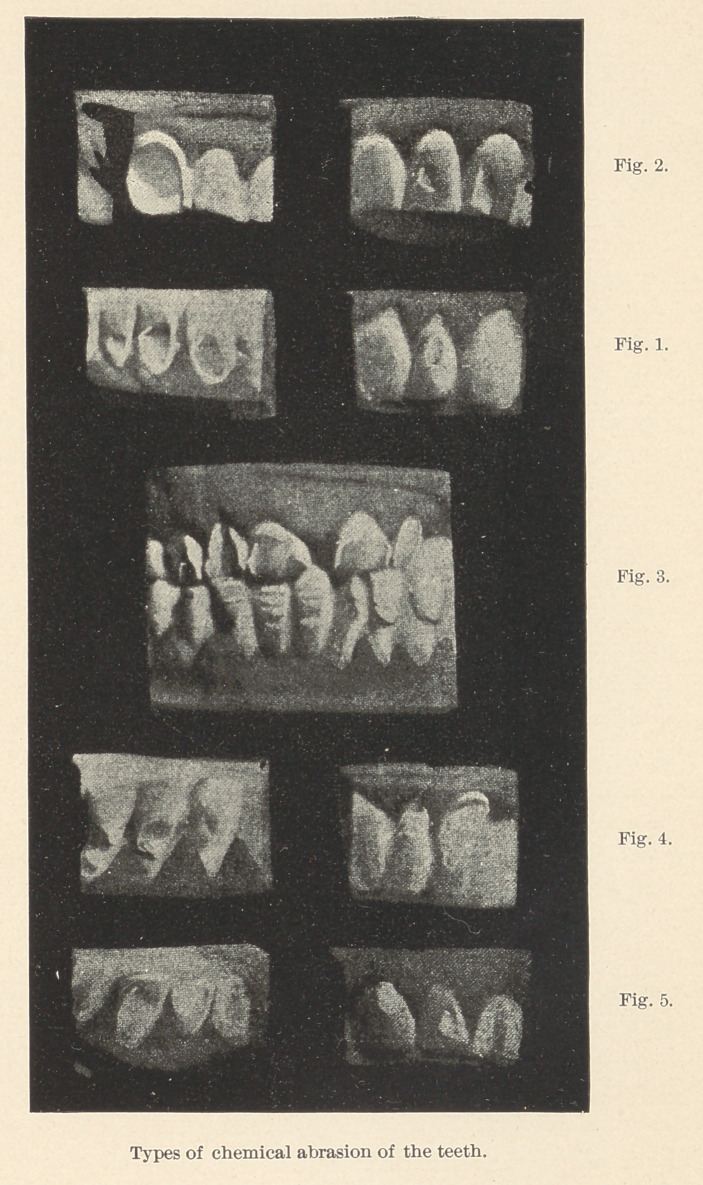# The New York Institute of Stomatology

**Published:** 1900-04

**Authors:** 

**Affiliations:** The New York Institute of Stomatology


### Reports of Society Meetings.

### THE NEW YORK INSTITUTE OF STOMATOLOGY.

A REGULAR meeting of the Institute was held on Tuesday evening, January 2, 1900, at the office of Dr. Louis C. Leroy, No. 6 Lexington Avenue, New York, the President, Dr. E. A. Bogue, in the chair.

The minutes of the last meeting were read and approved.

*The President.*—As we have so much of importance on hand to-night, we will omit communications on theory and practice, and proceed at once with two papers, the subject of which is the use of nitrate of silver in dentistry. Dr. J. Morgan Howe, who was to have read one of the papers, is unable to be present, but has sent his paper, which the secretary will read.

(For Dr. Howe's paper, see page 237.)

### DISCUSSION.

*Dr. S. H. McNaughton.*—While my experience with nitrate of silver has been quite limited, it has been on the whole very satisfactory. I believe that by its use alone I have in several cases made fillings unnecessary, and by its use in connection with fillings made

Fig. 2.

Fig. 1.

Fig. 3.

Fig. 4.

Fig. 5.

Types of chemical abrasion of the teeth.

more permanent operations and caused the dentine next to the plug to become less susceptible to attack by caries.

Of course the feature of its darkening the portion of the tooth to which it is applied, and sometimes much more than this, is an objection, and one which I fear may never be overcome, as when applied to superficial caries we expect no good results unless we have the characteristic stain; and if after a while we notice a loss of stain or a white spot appear as though the pigment were being dissolved away, we may be sure that the nitrate is not holding its own against the progress of decay.

For convenience in using I procure the salt in crystals, which I keep in a dark bottle; into this bottle with the nitrate a pledget of cotton saturated with water is placed, so that in a little time this becomes a saturated solution and is always ready for use. When I desire to use it I take a very small pledget of cotton or spunk on my pliers and dampen it with this saturated solution. I take away any surplus of the solution, as by so doing it is less apt to touch parts where it would do no good and might make an unnecessary stain.

Of course this stain is due to some black precipitate. I am not prepared to say just what this is. Bartley (1898) says, in speaking of silver nitrate, "On exposure to the light in the presence of organic matter its solutions turn black and precipitate metallic silver." Roscoe (1896) says this salt (silver nitrate) undergoes decomposition when exposed to the sunlight in contact with organic substances, and a black substance of unknown composition is formed. But whatever this substance be, it is exceedingly insoluble.

From its solutions metallic copper precipitates the silver as a fine powder; by mercury the silver is thrown down in arborescent form; gold and platinum cause no precipitation. If a pill of tin-foil be placed in a solution of the nitrate, it will become black and break into pieces, settling to the bottom as black powder and flakes.

In the application the action is not always the same. Sometimes there will be minutes before there is a perceptible change; at other times the precipitation will appear as quickly as though black varnish had been applied. Applied to dentine which has been previously stained by an amalgam filling, instantaneous staining takes place.

In one case where the teeth showed the greatest tendency to decay that I have ever seen,—application of it to a cervico-buccal cavity,—it not only became black at the place where the nitrate was applied, but the discoloration (not so much, of course) extended over nearly the whole side of the tooth. In another instance, where an anterior approximal cavity in a lower molar had been prepared for a gutta-percha filling, it was intended to touch with the nitrate the cervical border, including the so-called vulnerable point, but some of the solution got under the enamel at this point and caused a discoloration to the depth at least of one-sixteenth beyond the excavation into what appeared to be fairly sound dentine.

The following are the principal conditions in which I use this salt: For superficial decay at the cervices of teeth. Under some circumstances the first indication of something going wrong with the teeth will be a whitish line along the tooth or teeth closely parallel with the gum margin, in some of the very bad cases being most noticeable on the buccal and lingual surfaces of the molars, both upper and lower. It appears as though some solvent had been at work and had dissolved any deposit which had been on the teeth, or had refused to allow any to remain in this particular territory. At first the only perceptible result is to clear the tooth or teeth and make them appear whiter. In a short time there will be a long narrow white line of disintegrated enamel. The nitrate applied to this will produce the characteristic stain darkest in the line of disintegrated enamel and shading off towards the occluding surface.

In cases like the above the conditions are favorable for decay, but the following case, I think, will show very good results from the use of the silver salt. Mrs. C., who has always taken the greatest care of her teeth, and who, since becoming a patient of mine seven years ago, has had her teeth examined about every two or three months. One of her regular visits should have been made in September, 1896, but owing to sickness she did not come until November, and then was quite despondent regarding her teeth, as she said they were going very fast and that one was quite loose. Examination showed a bad condition of the teeth at the edge of the gums, in some places the white line of disintegrated enamel, with slight pits here and there, and extending nearly around some of the molars, while on other teeth there would only be the clean surface spoken of before. The left central was quite loose; perhaps "springy" would give a better idea of its condition, as it seemed as though the walls had become thinner and less firm. There was a pocket on one side, and I found that I could put an instrument nearly to the apex. This was a condition which had developed since her visit in July.

She told me that she had had a very peculiar sickness; that for over two weeks not a particle of saliva came into her mouth. Her appearance, she said, would indicate mumps, but her physicians said it was not mumps. I have since seen the description of a rare disease, xerostomia, which means dry mouth, and which was undoubtedly the same disease from which this patient had suffered. Following this sickness were the conditions just noticed. It was what might be termed a "desperate case." Applications of the nitrate were made to all places where the white or bad line appeared, and now, after three years and a half, fillings have been put into two or three of these places, while applications were made to nine or ten teeth. Although not in any way treated with the nitrate, I might say that the pocket alongside the central has been closed, and that it would be difficult to find any trace of it now.

Another place where I use the nitrate is in all cavities of temporary teeth other than those of the incisors; if the decay be only superficial, touching with nitrate may suffice, or if the tooth is to be filled, apply to the cavity before filling.

Two bicuspids somewhat more than superficially decayed were treated with this salt three years ago, and, so far as I can see, are in a condition not likely to require fillings at any future time. Young children will sometimes have the superior molars quite badly decayed on the buccal surface. These cavities will be broad and shallow in places, with margins of chalky enamel, and it is difficult to know when to stop with the excavating, if, indeed, the choice is left with the operator, for these teeth are usually very sensitive and the patients not the ones with the greatest pain-bearing qualities. After such excavating as is possible, these should be touched with the nitrate of silver and filled with amalgam, zinc phosphate, or a mixture of zinc phosphate and amalgam, and good results may be expected. If the cavities are sufficiently deep, perhaps gutta-percha will do very well. Dr. Elliott once mentioned to me that he nearly always touches with the nitrate, roots which are to be crowned. I have done this, and believe it to be good practice.

Dr. Bethel and others have advocated its use in the treatment of pulp-canals by depositing the silver in the canals by the use of the electric current. I have only tried this method cnce, and then to a very small root which had given much trouble and had been unsuccessfully sealed several times. A single treatment was successful, although I have no idea that I deposited the silver on the walls of the canal to anything the extent that was done in the specimens exhibited before this society a couple of years ago, but I think I got a good antiseptic condition, and perhaps everything organic was embalmed.

*Dr. Charles O. Kimball.*—Gentlemen, I have a communication from our associate member, Dr. Hopkins, of Boston, which I should like to read:

"DEAR DR. KIMBALL,—I am sorry I cannot attend the January meeting of the Institute. I have used nitrate of silver in my laboratory experiments, and have found that it made the teeth much more resistant to artificial caries. Chloride of antimony will do the same without discoloring the teeth, but I doubt if it is safe to use it in the mouth. Possibly such care might be taken by an operator of great skill that no harm could come from its use. I am happy to tell you that I have just succeeded in producing artificial caries with a pure culture of the bacillus mesentericus vulgatis. The media was one per cent. glucose bouillon, and the time was nine months. There are many other forms, probably, that will do the same, and it will now be much easier to work them out.

"I believe this is the first *authentic* instance where decay has been produced by pure cultures of mouth bacteria, and I feel that it is an important step in advance. There is no secret about it. I should only be glad to find others working in the same line.

"Sincerely yours,

"S. A. HOPKINS.

"BACTERIOLOGICAL LABORATORY, HARVARD MEDICAL SCHOOL."

*Dr. R. H. M. Dawbarn.*—I have been particularly interested in the subject under discussion. I have no doubt but that this property of arresting decay is due to the antiseptic value of the nitrate of silver. The silver salts have always been placed almost at the top of antiseptics. A few years ago Dr. Miquel, of Paris, went carefully over a long list of the most effective agents for preventing putrefaction in bouillon. Surgeon-General Sternburg, U. S. A., subsequently repeated and verified most of his assertions. You may perhaps be interested to know what the five most powerful antiseptics are, in their order,—

1. Biniodide (the red iodide) of mercury, which will prevent putrefaction in a beef-tea preparation, 1 to 40,000.

2. Iodide of silver, 1 to 33,000.

3. Freshly prepared peroxide of hydrogen, 1 to 20,000.
4. Bichloride of mercury, 1 to 14,300.
5. Nitrate of silver, 1 to 12,500.

That these are very much stronger than carbolic acid is seen by the fact that the latter will prevent putrefaction in beef tea only when the proportion has become as strong as 1 to 333. Of course, as to the objection to the use of nitrate of silver, it is a double one, as the writer of the first paper has pointed out: first, discoloration, and second, pain. As to the discoloration, when we use the nitrate we are in reality using indelible ink, and its internal use in the fields of medicine and surgery has for this reason been limited for many years. I do not know whether any one here to-night has ever seen a "blue man." I have seen one; and they used not to be uncommon. The ghastly blue-black hue was given to the skin by the prolonged use of nitrate of silver internally, in this case to cure epilepsy, a method no longer employed. The color differs very markedly from the brownish black of the negro. Now, the suggestion which I would make here is that you follow in the footsteps of the other practitioners of surgery and substitute for the nitrate the more powerful iodide of silver, which stands next to the very head of all antiseptics, as just stated; or else that you try some one of the newer salts of silver which have been brought forward for this very reason, to prevent staining and also irritation. We have the citrate, the tartrate, or may use one of the albuminates of silver,—argonin, for instance. These possess a somewhat similar antiseptic value to the argentic nitrate, but have not its discoloring properties.

*Dr. W. St. George Elliott.*—There is one point regarding the use of nitrate of silver which seems to me of great importance. Theoretically it is perfectly sound, but practically it is not yet proved. I refer to the thorough sterilization of root-canals by the use of nitrate of silver in connection with the cataphoric current, by introducing a solution of the salt into the bulbous portion of the canal and then carrying it through the canal by means of the current, and afterwards removing the *débris*. We know that by the aid of cataphoresis nitrate of silver may be carried through the entire length of the canal and as much farther as we may choose to carry it. If the case is then dismissed and seen the next day, the contents may be removed without the likelihood of further trouble.

In regard to the lactate of silver, I have used it for one or two years, but so far as my experience goes I have found nothing like the same results as with the nitrate, which latter I use in every cavity which is not exposed to view. There is an objection to the use of the nitrate of silver to be followed by an amalgam filling, as that filling will always have a black ring around it, and if seen by a dentist who is not familiar with the work, he may remove the filling, thinking it imperfect. Dr. Taft once said, "The stain of nitrate of silver is very superficial and easily removed." This, however, is not always the case. I am reminded of a case I saw in London a few years ago. The patient had a prominent canine, and the dentist, who, I am ashamed to say, was an American, instead of drawing it in, cut it down one-eighth of an inch with disks. The result was extreme sensitiveness, beyond the ability of the patient to stand. She returned to the dentist, who painted it with nitrate of silver. This had been done many years before, but the tooth was still much discolored.

*Dr. L. C. Leroy.*—In this connection I wish to call attention to the value of chloride of zinc as an obtundent, both in cases of abrasion and sensitive cavities. I have had excellent results, and it does not discolor tooth-structure.

*The President.*—I take great pleasure, gentlemen, in presenting Dr. Flanagan, of Springfield, Mass., who has kindly consented to read us the paper of the evening.

(For Dr. Flanagan's paper, see page 245.)

DISCUSSION.

*Dr. Elliott.*—This is an exceedingly broad question, and at the same time it does not seem to me at all a difficult one to understand. While I sympathize very fully with the speaker in his desire to awaken in the profession charitable instincts, at the same time there is a great deal to be said on the other side. That the poor are entitled to our best attention goes without saying. This fact has probably been better recognized in other countries. In England particularly a great deal has been done, far more, I fear, than will be done here in a generation. There it is quite the custom to appoint a dentist to all the public institutions. In some sections in England the school children are examined, prior to their entering upon their course of study, by a competent man who gives them a chart and insists upon their going to a dentist and having their teeth properly cared for before they are allowed to enter the school.

I have, as I suppose is the case with all the gentlemen present, received a large number of gratuitous patients, but something ought to be done in a more general way. I am sorry to say that I had very little success in this direction. In speaking to some of the staff of St. Mary's Hospital, I asked, "Is it not true that in most of your children the dental organs are in bad condition? Is it not of the utmost importance that the child should have proper dental organs for the mastication of food?" Of course, these gentlemen admitted this, but it took six months before sufficient funds could be raised to procure a suitable outfit for the hospital. I took up the work and for two years carried it on, spending an afternoon every week there. Ninety-five per cent. of the children had very bad teeth; in many cases not a single sound tooth. Many were suffering, and there were many cases of abscess. They were, indeed, in a pitiable condition. I appealed to those in charge of the hospital. I tried to meet the directress, but failed. I thought I had done all I could. My impression was that they should take a dental student and put him in charge of the children's teeth, paying him a reasonable amount, which need not be large. Nothing, however, was done. The instruments are still there. No successor has taken my place. Six or eight months have passed, and there the matter remains.

I have spoken to many physicians on the subject, and they all seem to recognize the necessity. The average physician does not know anything about dentistry. At one time I was surgeon in charge of the Ninth Army Corps Hospital. We had six surgeons, and not a single one who could treat an ordinary case of dental abscess. I myself must plead guilty to the same thing. I always relegated those cases to the hospital steward, and the only dentistry he did was extraction. It is true that there are men appointed to some of our institutions. I know of the dentist who has charge of the children in the Juvenile Asylum. I am told that all the dentistry done there, as in most of our public institutions, is extracting. Now, it does not require a great amount of talent nor a great amount of skill to treat children in our public institutions and do all that is necessary to be done, and this work could easily be done by students. It is very plain to be seen why our public institutions are not supplied with the best dental as well as medical skill. The medical practitioner requires a much shorter time to treat his patients than does the dentist, and for this reason he need never spend more than half a day in his office, thus leaving at least a balance of time for hospital work. The dentist, whose operations are long, must spend his entire time at his office. It is not that we are less charitable, but it is out of the question for us to close our offices often to attend a clinic. Something else must be done, and the only solution I can find is the employment of students.

*Dr. Leonard Weber.*—In looking over the abstract of Dr. Flanagan's paper, to which we have just listened with interest and pleasure, I noticed at once the importance of certain questions raised by him, and did not hesitate to accept your invitation to take part in the discussion.

To understand and practise the healing art of diseases of the teeth, gums, and alveolar processes alone, as a specialty resting on the basis of anatomy and pathology, means a good deal more than simply filling and pulling teeth. This has long been recognized by your leading men and colleges, and the requirements of the modern students of dentistry in the knowledge of anatomy, physiology, and pathology have grown in proportion to the demands of broader culture for the practice of this profession.

The instant we go beyond these narrow lines of dentistry, as understood up to the present time, we enter upon the broad field of general medicine and surgery, and it seems to me beyond question that an accomplished stomatologist will be forced to have as much education in general medicine as, for instance, a practitioner of otology is expected to have; that he must be, in fact, a medical man, who practises stomatology as his specialty.

Now, as to the first question, Are the practitioners of dentistry in the habit of doing any charitable work? Certainly, but rather individually, sporadically, and in connection with their own schools have they done so thus far. The immense field of public charitable work, however, they have scarcely entered. Why not? Because they have not been asked to come in. And why have they not been asked? Because they have not come as an organization before the various boards of trustees or directors of dispensaries and hospitals and offered to do the work that belongs to them, and said that they were ready to do their share and insisted upon getting it.

Go the rounds of dispensaries and hospitals, and you will readily find out that the sick poor—who are not in the habit of going to the dentists for obvious reasons—suffer not only from carious teeth that need filling, or stumps that need to be removed, but from various consecutive disorders, such as ulcerative gingivitis, fistulas, abscesses, facial neuralgias, phlegmonous inflammations, dyspeptic conditions, and, as we now know, from various autoinfections caused by the action of putrid material and pathogenic germs which may and do enter the general system from diseased foci of the mouth, just as from the gastro-intestinal tract.

Now, good dentistry applied in time to these cases is the best preventive medicine, better than rhubarb and soda to soothe the stomach or chinin to treat a neuralgia of this kind. And to cure such patients without the dentist's help would be asking to remove an effect without trying to remove its cause.

Out of many instances to the point which I could cite, let me give you only one, and a rather serious one, too. About three years ago a servant girl, twenty-four years old, apparently robust and without hereditary taint, came to me, saying that she feared she was going to be ill again as she had been two years before, when she lost a good deal of blood from the mouth and had to stay in hospital for about three months. On examination a case of hemorrhagic purpura—*i.e.*, Welhof's disease—was disclosed: bluish-red purpura and petechiæ over a great part of the body, blood oozing constantly from gums and mouth, scarcely a sound tooth in it, the gums in an advanced state of ulceration. Her teeth and gums had been sore for years. No one had advised her to go to a dentist, and if they had, she had not the money to pay for extensive treatment. Knowing from other experiences that purpura often is of infectious origin, I believed that the infection in this case came from chronic ulceration and inflammation of the gums. I had the patient in hospital about two weeks, just long enough to use the requisite local antiseptic and general supporting treatment, and then put her in the hands of a good dentist. She recovered entirely, was soon well and strong again, and has remained so ever since. When I related this case before a medical society I was not at all surprised to learn of similar experiences on the part of some of the members present.

Going back many years, when I did my share of dispensary work down at the Northern Dispensary,—from 1865 to 1870,—our apothecary there had assumed the task of pulling teeth. He was not very bad at it, but was awfully glad when I offered to relieve him of this particular duty. I know at the present moment of but one of your profession who holds a regular appointment as dentist to a large and well-known dispensary in this city, but he is paid for his services by the month. He is not a doctor of medicine, and does not belong to the Medical Board of the dispensary. When you gentlemen get together and establish some central plant of your own to do your public charitable work, which, I presume, you will try to do with the financial help of some of our ever-generous fellow-citizens, and more or less money which you have to put in yourselves, you may call the institute what you will think best, and go into the treatment of surgical diseases of the mouth as far as may be right. When you come to ask for appointments in dispensaries and hospitals, I believe you have to come in as practitioners of dentistry, unless you are graduates in medicine. We physicians are not hard to get along with. Though some of us, perhaps, are playing too much "dog in the manger" at times, the majority are liberal, but they are a bit jealous and suspicious in having titles created for new and comprehensive departments in this latter-day specialization of medicine. Provided you have a previous understanding with the medical boards of the various charities before you apply to the boards of managers, no serious opposition will be encountered by you in your laudable and timely enterprise. The public will be with you, of course. They love you fully as much as they do us; though they may not always like your bills, they are proud of your work. Many of them show it, too, and will naturally be delighted to get something good and useful for nothing. From all view-points it is a good thing you want to do; it is timely, also, and what is good and timely will succeed.

*Dr. D. B. St. John Roosa.*—I may as well say, in the beginning, that, except for a brief conversation with the President and with the speaker who has just preceded me, I have not prepared myself for the discussion this evening. I believed that I should find ample material for my part of it in what might be said before my own turn came, and I am not disappointed. In the first place, I am very much impressed with the fact that when professional men begin to investigate, or hear the results of such an investigation, into any other profession than our own, we very soon find that the world is just as bad in that direction as it is with us. In other words, we find that neither their ideal nor ours has been reached. And we find that we are living in a world where it is constantly necessary to keep the weeds out, so that the flowers and the useful things may grow. The dentists have reached the point when, perhaps, they think that the weeds are choking the flowers pretty badly. This may be so, but when I think how much each one of us here to-night, outside of your profession, is indebted to your successful labors for his capacity to eat and digest his food; when I think of the high position of dentistry, especially in this country, I wonder very much that you remain, for some cause or another, with a great wall between yourselves and ourselves, who are members of the medical profession, although in many instances also specialists.

As regards the necessity of your presence in public medical institutions: Suppose any dentist came down to the Post-Graduate Hospital, or the Manhattan Eye and Ear Hospital, with both of which I have relations, and said, "Why, many of these patients, the young people especially, are suffering from their eyes or their ears or their stomachs because their teeth are in an absolutely fatal condition to perfect health." Now, we will all admit this, but what are we to do? We cannot hire dentists to come down and take charge of these cases, and they will not come without being hired. One gentleman here has practically told us that it is impossible for one of your profession to make a living without working from ten to twelve hours a day. I do not believe it is necessary or wise to spend so much time in your offices. I know that I would not be living to-day if I had devoted ten instead of five or six hours a day to practising my profession in an office. Your fees may not be large enough. The sooner you make the people believe that the teeth are just as important as the ears or the eyes, the better it will be for you and for mankind. In my judgment you have not, with your fees, reached any conception of your capacity and skill. You work too much as mechanics, too little as surgeons.

You know how dentistry began in this country. There were certain practitioners of medicine who began to study the surgery of the teeth, and in due course of time they began to practise dental surgery exclusively. I think it was in Baltimore that the first organization of American dentists sprung up, and at the beginning dental surgeons were medical men. Why did dentists ever depart from this position? It is not such a very difficult thing to get a medical degree; and I think the fundamental trouble is right here, that dentists do not insist on their successors getting the degree of Doctor of Medicine. Of course, all this general knowledge is not important in one sense in your profession, but in a certain sense it is of the greatest importance. It gives men a general conception of the human body, which prevents them from getting rattled when anything happens beyond the teeth. There is no reason why dentists should be outside of the medical profession any more than ophthalmologists or otologists. We need you in the medical profession, in our hospitals and colleges. The separation is absolutely unnecessary and illogical, and it cannot continue. At the Post-Graduate Hospital we have a dentist who, I believe, is also a graduate of medicine. He is attached to Dr. Powell's clinic, and he is of the greatest service. He does not practise general medicine, but he does first-class work.

When I had the honor to be a member of the Council of the New York University I became interested in seeing a dental department established, each graduate from that department to have the degree of M.D. Although I had the full sympathy of the board, there was a general indifference on the part of dentists and bankers, and between the two the scheme came to nothing. But I am as sure as I am of anything which I can simply foresee, that it is only a matter of time, and perhaps a very short time at that, when we shall all be together again. I say "again," because we began together.

Mr. President and gentlemen, it is a very great pleasure for me to be here to-night. I remember attending two or three years ago, when you were kind enough to invite me, and I was impressed with the scientific merit of your discussions and papers. You should deal with this problem also in all seriousness. You must, in order to be professional, do a certain amount of charity work, and public charity work. Far be it from me to insinuate that you do not do charity work in your private practice, but the sooner you engage in public charity work the better. The sooner it is understood that in our infirmaries good advice may be had for bad teeth as well as for bad legs or bad lungs, the better it will be for the health of this great community. I have no doubt there are men who will give you money when they see how serious you are about it, and also that the government of the United States will take care of the teeth of its army and navy if properly approached. At any rate, we are not so badly off in this country, in this respect, as in one other country. Have you read in the *British Medical Journal* of the recruits who were rejected on account of their bad teeth, and finally had to be accepted, bad teeth or not? Have you looked seriously to see the whole of England growing up with bad teeth? Even noblemen have bad teeth in England. Fine ladies have bad teeth in England. Our American woman thinks it a reflection upon herself to have bad teeth. So let me advise you to go ahead with your charity work.

Establish in this city proper places where such work can be carried on, and the rich men of New York will be glad to pay for it.

*J. Cleveland Cady.*—I fancy that the professional men before whom I have the honor to speak this evening have reached their high standing by means similar to those which an accomplished skater availed himself of to attain his proficiency. Said the young lady who had been looking on with rapt admiration, "Why! why! as you have been skating so gracefully you have cut your monogram on the ice! How could you do it? How did you acquire such an accomplishment?" "Oh," he replied, with a sigh, "it took a great many sittings."

Through "many sittings" we gain valuable experience as we pass on in life. How often we wish for the opportunity to live life over again, that we might profit by our acquired knowledge! But though we cannot retrace our steps, we can turn the light of our experience on the paths of our younger brethren, so that after all our many sittings shall not be lost.

The alluring object before most young practitioners, whether of dentistry, medicine, or other callings, as they set forth in life, is to get a little clientele, productive of a moderate income, which shall give many comforts and pleasures, perhaps even luxuries. Later this ambition expands and includes means for support of a wife and a modest home; still later an advance that shall give influence, and with this there presently springs up the desire to become rich; and if he is a bright, pushing man, bent upon wealth, he probably will attain it, and in all likelihood live and die a commonplace man, in no respect an admirable character, or one whose departure from the stage of life is greatly mourned.

The life of the average practitioner is not an inspiring one unless something has entered into that life,—outside of itself,—its personal aims and ambitions. Recall in your own profession those whose careful and conscientious judgment you most respect, the sincerity and weight of whose character you most admire, and you will invariably find that they are the characteristics of one whose life is not self-centred, but whose heart and efforts have gone greatly outside his personal interests.

In some cases, where their own professions have offered no particular openings for beneficent work, they have found that which their souls longed for in the work of some of the various organizations and boards for relieving the unfortunates. They have interested themselves in the children of the poor as they met them in the mission schools; they have served efficiently on hospital boards, or in the work of prison reform, or perhaps in the cause of municipal betterment, and personally have found what their hearts craved, the uplift their souls needed, but their own profession has not been honored to such an extent as it would have been had their benevolent work been more directly connected with it.

Dr. Watson's touching portrayal of the two doctors, MacClure and Sir George, in "The Doctor of the Old School," that touching tale in "The Bonnie Briar Bush," decidedly elevated the profession of medicine in the minds of all who read it, and inspired many a young man with a noble ambition. People of wide observation know that there are many Dr. MacClures in our own country. Let me speak a moment of one who not long since went to receive the eternal reward of "a good and faithful servant." I refer to Dr. Love, of Montclair.

I had often heard of him in connection with the admirable little public hospital he founded in that place, with no small effort and sacrifice. Those connected with it often told me that the whole town loved and honored him, and that from his life the word "physician" had gained a significance in that place that it had not elsewhere. One day as I was lunching with a well-known public man, whose home was in Montclair, I happened to speak of the good doctor, and he responded, with no little feeling, "It seems to me everybody in the place is a debtor to him, and none more than I. After the birth of my last child my wife did not rally well; for months she was greatly prostrated and depressed. It seemed to me that he kept her life from going completely out. With his great heart he encouraged her and cheered her. He really bore her up till at length the change for the better began, and her health was regained. We owe everything to him."

Dr. Love's death was a most singular and beautiful termination to a life whose passion was for rendering service to those needing it. He was now an old and venerable man, yet attending his duties as fully and closely as ever. One day he had performed a most difficult and trying operation for a poor woman, a charity patient. It was the turning-point of life or death, and his skilful operation was a saving one; but as life was secured for her the wearied old physician sank back, his eyes closed, and his blessed ministries ended forever. The town was in tears and mourned for him as a father.

Some months later Ian Maclaren read to a large audience in the place his story of "A Physician of the Old School," of the noble and heroic work of Dr. MacClure. As he proceeded with the story a solemn and almost painful hush came over the audience, and its eyes were filled with tears. Said the narrator, "It was not Dr. MacClure whose image stood before them, but Dr. Love!"

I am sure you will agree with me that the spirit of such a life is something we all have reason to covet and earnestly seek, and that it is positively essential to any true life, to any worthy exercise of our powers, and if we cannot find opportunity for it in the lines of our own profession, let us by all means go outside, and find and exercise it somewhere and somehow.

But if one has an honorable pride in his calling, and longs to see that calling more and more esteemed, he will seek to make his profession a benevolent one, a generous one; one which without reward seeks to bring mercies and blessings to those in want; one which is quick to respond with a large and warm heart to the cry of human need; that knows by experience the spirit of Him who taught by His own example as well as by precept that it is more blessed to give than to receive. This spirit and rule exalts and glorifies any calling; without it the noblest profession becomes a mere mercantile pursuit.

*Dr. Kimball.*—There is but little that I can add. As I have listened to the remarks of our friends, Drs. Weber and Roosa, I have felt that they were striking at the root of the matter, the need of a higher education for dentists and a closer relationship of dentistry with its fellow, general medicine,—really the need of the day as far as our profession goes. I need not tell you, Mr. President, nor you, my fellow-members of this Institute, how close this has been to my own thoughts and to the thoughts of all those who have been associated in this Institute. Perhaps it may not be unfitting here to say that we, as an Institute, are even now taking steps to see if we cannot begin a definite charitable work, in connection with our organization, for the benefit of the city; a work which it is proposed to put upon the sound footing that is the basis of all wise hospital work as I understand it,—viz., that the profession benefits the patient, and that the patient benefits the profession in return for the services received, thus bringing about a better knowledge and a higher degree of skill on the part of the practitioner. This, I believe, is the basis upon which all hospitals are conducted. The work is not merely a work of healing, but a work of education. They go hand in hand. It seems to me that it is a necessary relationship, so that any infirmary or dispensary work which does not carry with it this idea of the betterment of the profession at large is radically faulty, looked at from the view-point of the benefits to humanity at large. There are details connected with this plan to which I need not allude. Dr. Elliott has mentioned the fact that it is difficult for the practising dentist to give the same amount of time to this work that the physician can. As we know, the physician can treat a case in a very few moments and with great skill, whereas the dentist has not only to give advice, but to go through a long mechanical operation which takes a great deal of time, even for the simplest things. Nevertheless, facing this problem and its difficulties, I may say that when our plans are fully carried out we expect to be able to undertake some such charitable work here in this city. I trust the time may not be far distant.

*Dr. Leroy.*—I would be only too pleased to contribute my services to a work of this kind. I endeavored to do this at the Northwestern Dispensary, but I find that for one man so much time is required that it is almost impracticable. I would suggest that we band ourselves together and contribute an hour at certain intervals.

*Dr. Elliott.*—I do not wish to be misunderstood in this matter when I spoke of the difficulty of giving time to this work. I am sure that with all my heart I would like to see some practical way develop in which this can be done. I will be very willing to give half a day a week to such a purpose, and I think if we all enter into the work there will be no difficulty. But we must not enter into it without due consideration. Many of us remember the dispensary which was started in Brooklyn, and which gradually dwindled away. I sincerely hope that this thing will be undertaken, but it must be done in such a way as to make a permanent success of it.

*Dr. J. G. Palmer.*—Dr. Leroy has brought to my mind the fact that a good many years ago, when I was a student with Dr. Streeter, I was connected with the Northeastern Dispensary, but the trouble was that they only wanted teeth extracted and nothing else done, so my work did not continue long.

Dr. Weber's remarks also brought to my mind the fact that one of our prominent associate members has been asked to accept a position as dental surgeon to the Hospital of St. Mary's, at Passaic, N. J., to be made a member of the regular staff without receiving any salary, which position he accepted and is fulfilling the duties thereof. Regarding representation in the army and navy, it has seemed to me that if all of us would bring a little more pressure to bear, we might secure this representation. Dr. Roosa mentioned the fact that we should raise our fees. In this connection I call to mind the case of a physician in a New Jersey town who insisted upon his daughter going to Philadelphia to have her dental work done, because there were so many dental colleges there, and she could get it done for nothing.

*Dr. Charles A. Meeker.*—I believe the explanation of the whole secret comes from the medical side to-night,—from Dr. Weber. I think we should go as an organization to the trustees of these institutions. I trust this discussion will start the thing going in the right direction.

*The President.*—Possibly Dr. Weber does not remember the exact condition of things in Baltimore which about fifty years ago led to the separation of dentistry in this country from the general instruction of medicine. We were at that time informed by our medical friends that dentistry had nothing whatever to do with medicine, and, thus having been thrown off, we were obliged to shift for ourselves. This necessarily resulted in the establishment of a dental school, and from this beginning have grown all the institutions of that kind which dot our land. The essayist of the evening, alluding in the course of his remarks to interdental splints, has recalled to my mind quite forcibly one or two little incidents. An old lady fell down stairs and broke her jaw. The surgeon who undertook to set it had never had a case like this with no teeth in either jaw. He did not know what to do, and it never occurred to him that he might call a dentist and consult with him regarding an appliance. Instead he made a plaster-of-Paris splint, in applying which he thought it necessary to remove her artificial teeth. There was a ligamentous union only, and the good old lady died soon afterwards. The unfortunate surgeon did not think to put her artificial teeth back where they came from after knocking off a few of the front teeth, and then bandaging the jaws together. Another surgeon of national eminence, who has gone to his reward, had a case of something in the antrum, which he prepared to open externally. A dentist was called in to see the operation, and just before beginning, when the patient was anæsthetized, the dentist took an exploring instrument and, passing it up through the socket of the tooth, engaged a bit of root which had been forced through into the antrum, and showed it to the surgeon, who immediately removed it without the aid of the knife. An eminent surgeon was called to my house to see a lady who, from an impacted wisdom-tooth, already had half a dozen openings on her face. The surgeon lanced the abscess transversely to the striæ of the upper part of the temporal muscle, and as the lancet went across instead of parallel to the muscular fibres, there was a scar. I mention these cases simply to show the urgent need that there is of some harmony of work between all branches of the medical profession. I do not recognize dentistry as a profession; it is a specialty. I do not speak of what is called mechanical dentistry. It is only about two years ago, I think, when I had the pleasure of presenting at the Surgical Section of the New York Academy of Medicine an appliance invented and made by our fellow-member, Dr. Michaels, a dentist of Paris, for replacing the upper third of the humerus, including at the same time the whole of the shoulder-joint, the upper end of the apparatus being screwed to the scapula. This apparatus was inserted and worn for a year and a half. Little by little new bone was formed, and the apparatus was finally extracted, and the man to-day has a good arm. The same dentist made an artificial lower maxilla, which was inserted and worn until the death of the patient, which occurred a good while afterwards.

Dr. Roosa spoke of the bad teeth in England. It is true that lords and ladies have bad teeth there, almost as many bad ones as they have here, but at the same time the science of dentistry has made such progress abroad that we will do well to look to our laurels.

*Dr. Flanagan.*—I cannot tell you how pleased I have been with the discussion of my paper, and in reply I trust I will be excused if I get a little personal. Charitable work in connection with hospital and dispensary has been mentioned several times this evening. Now, all professional men may be divided into two classes, those who are looking to the pecuniary end, and those to whom the pecuniary part is a secondary matter. Why, gentlemen, it is not necessary to have a dispensary in order to do charitable work. It is not necessary to go into a hospital. It is not necessary to have the degree of M.D. You have in this city institution after institution where your services are needed. Suppose you give but one hour a week, that is fifty-two hours in the year. But what is needed is organization in this work. I have the utmost confidence in this Institute of Stomatology,—and what more ennobling work than to be the pioneers in organized charitable dental work?

In closing I want to thank this society for its courtesy in asking me to come here to-night. This is a matter which is of great interest to me, and I have been awaiting an opportunity to present these thoughts to a society such as yours. I am at present engaged in this work in my own city. I am in New York now to buy a dental engine to be used in an institution where we have been doing this work, using a dollar-and-a-half easy-chair and carrying our engines back and forth. I trust that I have done some good, and that this work may be taken up by your society.

*Dr. S. E. Davenport.*—I think the Institute has been greatly honored this evening. We owe our thanks to Dr. Flanagan for his very interesting paper; also to a number of very busy gentlemen who have shown their interest in this subject by accepting our invitation to be present and discuss it. I move that a vote of thanks be extended to all of these gentlemen.

Motion seconded and carried.

Adjourned.

Fred. L. Bogue, M.D., D.D.S.,
*Editor The New York Institute of Stomatology.*

## Figures and Tables

**Fig. 2. Fig. 1. Fig. 3. Fig. 4. Fig. 5. f1:**